# Comparing Microleakage in Root Canals Obturated with Nanosilver Coated Gutta-Percha to Standard Gutta-Percha by Two Different Methods

**Published:** 2011-11-15

**Authors:** Yazdan Shantiaee, Farzin Maziar, Omid Dianat, Faranak Mahjour

**Affiliations:** 1. Department of Endodontics, Dental Research Center, Dental School, Shahid Beheshti University of Medical Sciences, Tehran, Iran.; 2. Endodontist, Private Practice, Tehran, Iran.; 3. Department of Endodontics, Iranian Center for Endodontic Research, Dental School, Shahid Beheshti University of Medical Sciences, Tehran, Iran.; 4. General Dentist, Tehran, Iran.

**Keywords:** Dental Leakage, Endodontic, Gutta-Percha, Root Canal Filling Materials

## Abstract

**INTRODUCTION:**

Favorable apical seal of root filling materials is a crucial factor for a successful root canal treatment. The aim of this in vitro study was to compare bacterial and dye microleakage of two root canal filling materials including standard gutta-percha and nanosilver coated gutta-percha, and to evaluate the agreement between results of these two methods.

**MATERIALS AND METHODS:**

Fifty-eight extracted single-rooted teeth were randomly divided into two experimental groups of 26 each, and two control groups of three each. After decoronation, root canals were instrumented by crown-down technique. Obturation was conducted using standard gutta-percha in one of experimental groups and nanosilver-coated gutta-percha in another group. AH26 sealer was used as the sealer in both experimental groups. Bacterial leakage was investigated after 60 days using Enterococcus (E.) faecalis microbial strains, and dye leakage was assessed during 72 hours using 1% methylene blue. The data were statistically analyzed by Chi-square test, Kaplan-Meier survival analysis, and Cohen’s Kappa.

**RESULTS:**

There was 84% bacterial leakage in standard gutta-percha group and 76% in nanosilver gutta-percha group. Complete dye leakage occurred in 24% and 27% of standard and nanosilver gutta-percha groups, respectively. The above difference between groups was not significant. In the samples with leakage, recorded times of leakage were not significantly different. There was no significant measure of agreement between dye and bacterial penetration along root-end fillings.

**CONCLUSION:**

There was a poor agreement between dye and bacterial leakage methods. Leakage results produced by nanosilver gutta-percha were comparable to those by standard gutta-percha. Considering the antibacterial effects of nanosilver coated gutta-percha, use of this type of gutta-percha might be more efficacious in endodontic treatments.

## INTRODUCTION

Microorganisms are major etiological agents in pulpal and periapical diseases [1][2]. Therefore, well-compacted root filling material with good apical seal and no leakage are crucial factors for a successful root canal treatment [3]. Dye leakage and bacterial leakage are the two most common methods used for assessing leakage of root-canal fillings [4].

Different materials have been used in endodontics as root canal fillings. Gutta-percha is one of the most prevalently used materials which have been used as a root-filling for over a century. Gutta-percha has been proved to demonstrate slight antibacterial property, mainly attributed to the zinc oxide in its components [5]. However, this does not render gutta-percha as an effective microbiocide. While destruction of microbial pathogens is a key to endodontic success, filling materials should ideally have an appropriate level of antibacterial effect. In an attempt to upgrade the antibacterial effect of gutta-percha, Dianat and Ataie have introduced nanosilver gutta-percha, the standard gutta-percha that is coated with nanosilver particles [6]. The nanosilver gutta-percha demonstrates significant antibacterial effect against Enterococcus (E.) faecalis, Staphylococcus (S.) aurous, Candida (C.) albicans and Escherichia (E.) coli [6]. This material is also proven to be highly biocompatible [7]. Nevertheless, the effects that adding nanosilver particles to gutta-percha surface might have on the efficacy of apical sealing has not been investigated.

The aim of this in vitro study was to assess bacterial and dye microleakage of two root-canal fillings, standard gutta-percha and nanosilver gutta-percha, and to compare the agreement between results of these two methods. The behavior of the E. faecalis microleakage was monitored in this study.

## MATERIALS AND METHODS

### Preparation

In this study, 58 single-rooted anterior teeth were used. Before operation, radiographs of each tooth were taken, and the presence of a single canal, fully-formed apex, and the lack of internal or external resorption, crack, calcification, or root caries were confirmed. The teeth surfaces were cleaned with curette and were stored in 5.25% sodium hypochlorite for 1 hour. The teeth were then rinsed with and stored in normal saline. The crowns were removed and the root length of each tooth was tailored to 13mm. Each root was instrumented using the crown-down technique by Hero (Micro-Mega, Besancon, France) rotary files up to number 30. The teeth were irrigated with 5.25% sodium hypochlorite before and after recapitulation. All preparations and filling procedures were performed by one operator.

The teeth were randomly divided into two experimental groups of 26 each, and two control groups of 3 each. In the first group, the root canals were filled with standard gutta-percha (Ariadent, Tehran, Iran) and AH26 sealer (Dentsply, DeTrey, Konstanz, Germany) by lateral condensation technique. In this group, a standard gutta-percha master cone size #30, covered with freshly AH26, was applied within 1mm of working length. Additional fine accessory standard gutta-percha cones were placed using a NiTi #25 spreader starting 1mm short of the working length. The root canal was considered to be adequately filled when the spreaders could no longer penetrate beyond the coronal third of the canal. After root canal filling was completed, the excess coronal gutta-percha was removed with a hot instrument. The same procedure was conducted for the second group using nanosilver gutta-percha instead of standard gutta-percha. Three of the remaining teeth were left unfilled after preparation, and were designated as positive controls. Negative controls consisted of 3 teeth fully prepared, and filled by sticky wax in order to prevent bacterial leakage.

In order to avoid penetration of materials through dentinal tubules and accessory canals, external surfaces of all 4 groups were coated with two layers of nail polish.

### Bacterial microleakage test

Double-room technique was used for bacterial leakage measurement. The 1.5mm ends of three-millimeter micropipettes (Supa Co., Tehran, Iran) were chopped, and the tooth was located in the cap end part. Sticky wax was employed to fill in the space between tooth and micropipette. The teeth inside the micropipettes were exposed to ethylene oxide for 12 hours. 10mL of Tryptic Soy Broth (TSB, Merck, Darmstadt, Germany) was added to glass test tubes. Each of fifty-eight tubes that contained 10mL of TSB was autoclaved and incubated at 37ºC. The clearness of media was considered a guide for sterilization. Under the sterile hood and heat, the microtube root assembly was mounted inside the glass test tube with the root tip contacting the culture medium. Afterward, they were isolated with Parafilm (Supa Co., Tehran, Iran). The set was incubated at 37˚C for three days, and the clearness of the media confirmed sterilization.

E. faecalis (ATCC 29212) were obtained from Pasteur Institute, Tehran, Iran. In order to reach accuracy of bacterial purity, they were first cultured in specific media. Pure bacteria colony was derived from blood agar, and then cultured in 10cc TSB. The set was then incubated at 37ºC for 24 hours and a 0.5 McFarland (1.5×108 bacteria/mL) solution concentration was achieved. Using a sterile micropipette, 0.1mL bacteria were placed into the test tubes in contact with the coronal access opening of the filled roots every 5 days for a total of 60 days. Parafilm (Supa Co., Tehran, Iran) was used to seal micropipettes and 50mm of test tubes every time bacteria were added. The test assemblies were incubated at 37ºC, and the culture medium in the lower chamber was monitored daily for turbidity.

A microbial contamination was recorded when turbidity was perceived. Inocula selected from turbid tubes were spread on bile esculin under incubation conditions to verify the existence of E. faecalis. The day turbidity was perceived, was recorded.

### Complete dye leakage test

After 60 days, the samples were rinsed with chlorhexidine and distilled water, and then were dried. The specimens were completely immersed in 1% methylene blue solution (pH 6) and placed in a Pyrex desiccator jar. The jar was connected to a vacuum pump until the jar pressure was reduced to 800 mbar in 30 minutes according to Dickson and Peters study [8]. The samples were kept in the dye solution under vacuum for 72 hours. The teeth were removed from the dye solution and washed under water. Afterward, 1mm of the apical tip of the teeth was cut by disk. Dye penetration between root filling material and the tooth walls from the apical aspect was monitored with stereomicroscope ×40 (Olympus, Tokyo, Japan). Whenever the dye leakage was observed at the interface of root filling material and the tooth root at the cut point, complete leakage was recorded for that sample.

### Statistical analysis

The results were analyzed using the Chi-square tests. Kaplan-Meier survival analysis was used to assess the trend of leakage after 60 days. Furthermore, evaluating the agreement between the two bacterial and dye microleakage tests results were done by determining Cohen’s Kappa coefficient. P<0.05 was considered statistically significant.

## RESULTS

### Bacterial leakage

**Figure 1:**
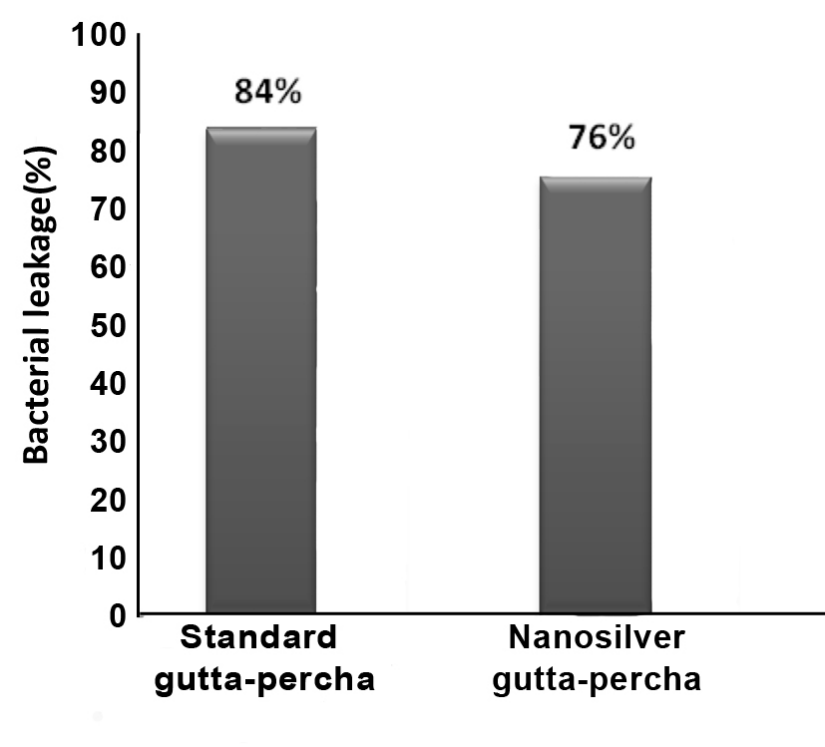
Percentage of bacterial leakage in standard gutta-percha and nanosilver gutta-percha at 60 days

Complete leakage was recorded within three days in positive controls. In contrast, negative samples demonstrated no leakage after 60 days. The results of bacterial leakage are shown in Figure 1 and Table 1. In the bacterial leakage test, the results demonstrated no significant statistical difference between the two experimental groups (P=0.48).

**Table 1:** Mean and Standard Error (days) of bacterial leakage in standard and nanosilver gutta-percha

**Group**	**Mean**	**Standard Error**	**Inferior limit**	**Superior limit**
**Standard gutta-percha**	19.28	4.29	2	60
**Nanosilver gutta-percha**	28.38	4.01	2	60

**Figure 2:**
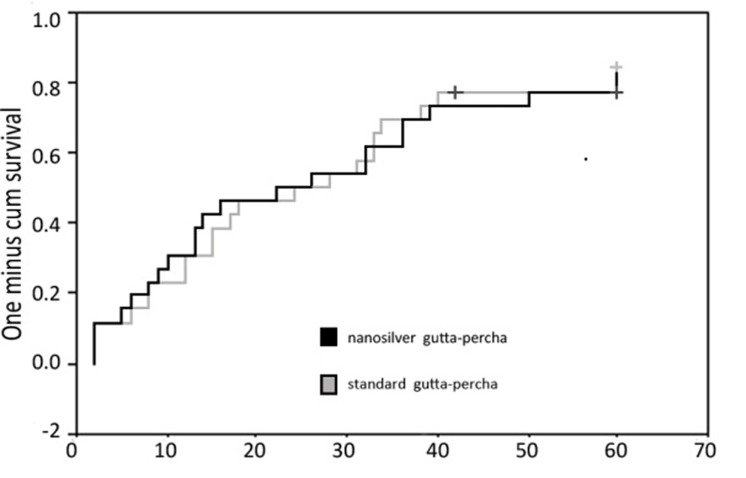
Trend of bacterial leakage after 60 days of experiment in standard and nanosilver gutta-percha groups

Time of leakage observation was recorded for each sample with leakage. No considerable difference was observed among the groups (P=0.75). Trend of leakage after 60 days of experiment was calculated by using the recorded times of leakage. No significant difference was perceived among the groups (Figure 2).

### Dye leakage 

**Figure 3:**
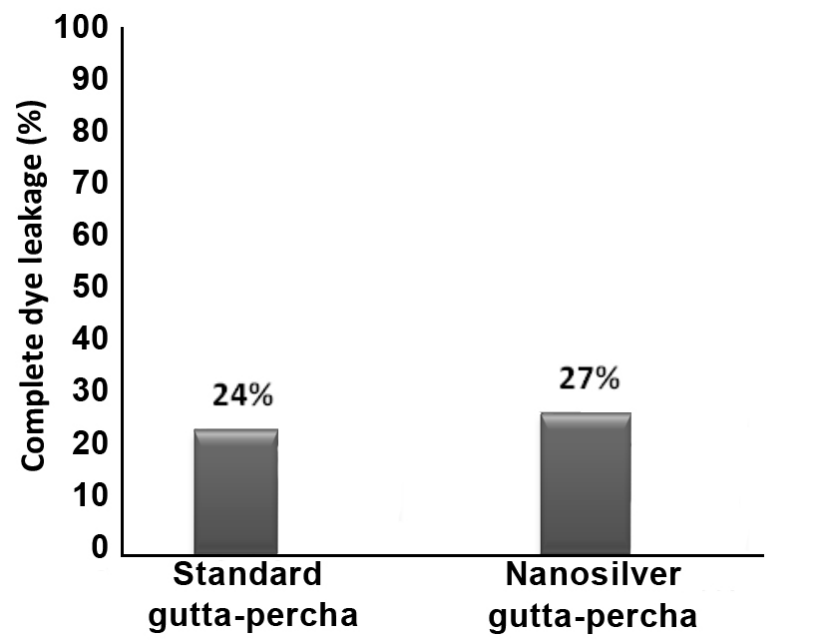
Percent of complete dye leakage in standard gutta-percha and nanosilver gutta-percha in 70 days

All positive controls showed complete dye leakage. In contrast, negative control group did not demonstrate any leakage. The results of bacterial leakage in each material are shown in Figure 3. The differences between groups were not significant (P=0.75).

### Agreement in leakage measurement with dye and bacteria

The comparison of the leakage measurements in dye and bacterial leakage methods was performed by inter-rater agreement (Kappa=0.088). While Kappa=0.00 was considered as poor agreement [9], no significant agreement was perceived between the results of the two methods.

## DISCUSSION

In the present study, the apical sealing of standard and nanosilver gutta-percha as root-filling materials was compared by dye and bacterial leakage methods. Duration of this study was 60 days, which was similar to other studies [10][11]. Applying two methods of leakage measurement enabled us to obtain more data and to compare the results of two methods.

No significant difference was perceived between two materials either in bacterial or in dye leakage results. Moreover, no considerable difference was observed between recorded times of occurrence of bacteria leakage for two materials. Considering the results of this study, it seems that, in terms of apical sealing, efficacy of standard gutta-percha and nanosilver gutta-percha as obturation materials are similar. Therefore we can conclude that nanosilver not only does not decrease sealing ability of gutta-percha but also increases its anti-pathogen properties.

Nanotechnology is one of the most promising fields for generating new applications in medicine, and nanosilver is one of products utilized for such applications. At nanoscale, silver exhibits significantly unusual physical, chemical and biological properties. Due to their strong antibacterial activity, nanosilver coatings are used on various textiles, bandages, wound dressings, ointments as well as coating on certain surfaces such as implants, catheters, prostheses and even washing machines [12][13]. Considering this wide range of applications, nanosilver coated gutta-percha was introduced in 2008 for the first time by Iranian researchers to prevent bacterial colonization in root canal space. One initial study implicated its antibacterial activity [6], and a recent study has confirmed its biocompatibility [7].

Different studies have been performed by using different bacteria [11][14]. In this study, E. faecalis, the main bacterium in chronic apical periodontitis and failed root canal treatment, was used [15][16]. The main reason that we employed cold lateral condensation technique for obturation was the near 90% success rate of this method as well it’s popularity [17].

In the present study, the results of two microleakage methods showed different apical sealing ability of experimented materials. Dye leakage method measures material adaptation along the canal walls, and since the size of dyes are small, this method may measure bacterial byproduct penetration. Bacterial leakage, in contrast, evaluates the sealing ability of all portions against bacteria. In our study, the bacterial leakage method showed greater microleakage than dye leakage method, and the agreement between two methods was poor. According to Barthel et al. study several reasons might have caused such results [18]. For example, some factors such as pH, temperature, or ionic charge, are all changed with bacteria, while cannot be simulated with dye method. Moreover, bacteria’s ability of active movement and growth, as well as ability to change shape and size, can result in more penetrations. Kersten and Moorer [19] have showed that the smaller dye molecules can penetrate more. As a result, it might be said that dye molecules, significantly smaller than bacteria, are capable of a deeper penetration. In addition, it has been reported that dye cannot completely penetrate between the sealing material and tooth, even after a given time. The presence of air bubbles that inhibit the penetration process is suggested as a reason for this fact [20].

These results corroborate with those of the previous studies that also attempted to compare different microleakage methods [18][21][22][23]. Other studies [21][24][25][26] have also found no correlation between leakage methods with various filling materials, concurring with our study. Barthel et al. applied the dye leakage test after the bacterial test on the same teeth and found no correlation between the tests [18]. They stated that such result might be caused by differences in working principles of various test methods or by the different nature of obturation materials.

Pommel et al. have also compared fluid filtration, electro-chemical and dye leakage tests for evaluating the sealing ability of single-cone and vertical condensation obturation techniques, and found no correlation among the tests [22]. They indicated that this result was not surprising because a correlation implied that leakage phenomena were governed by electrical, filtration and diffusion laws at the same time. It was stated that the outcome of the study depended on the test method.

Our results raise serious doubts about the information obtained by previous microleakage studies regarding the sealing ability of different endodontic materials. This also raises the question regarding the clinical relevance of leakage evaluation in-vitro. Thus, correlation is lacking between the sealing quality of root filings determined in-vitro and the tissue response observed in-vivo.

## CONCLUSION

There was poor agreement between dye and bacterial leakage methods. Therefore, bacterial leakage cannot be predicted by dye leakage results. It can be concluded that nanosilver coated gutta-percha provides leakage results comparable to standard gutta-percha which is commonly used as root filling materials. Considering the antibacterial effects of nanosilver coated gutta-percha, use of this type of gutta-percha might be more efficacious in endodontic treatments. However, further ex vivo and in vivo studies are needed to assess other properties of this new material.
